# Acceptability of a Large Language Model (LLM)-Generated Guideline-Based Checklist Among Otolaryngologists: A Cross-Sectional Survey and Thematic Analysis

**DOI:** 10.7759/cureus.106804

**Published:** 2026-04-10

**Authors:** Shahid Iqbal, Mobin Ahmadi, David Ahmadian, Peter Eskander

**Affiliations:** 1 Department of Otolaryngology - Head and Neck Surgery, University of California San Diego, La Jolla, USA; 2 Department of Acute Medicine, Royal Free London NHS Foundation Trust, London, GBR

**Keywords:** artificial intelligence, checklist development, clinical practice guidelines, evidence-based clinical guidelines, large language models (llm)

## Abstract

Purpose

Large language models (LLMs) have been useful for synthesizing clinical practice guidelines into decision-support tools; however, their utility for clinicians has not been formally evaluated. This study aims to generate a structured clinical checklist from an otolaryngology guideline using an LLM and to assess clinician perceptions of its accuracy, usability, safety, and likelihood of adoption.

Materials and methods

An LLM (ChatGPT version 5.2, OpenAI, San Francisco, CA, USA) was provided with the American Academy of Otolaryngology-Head and Neck Surgery Clinical Practice Guideline: *Evaluation of the Neck Mass in Adults* and instructed to generate a concise checklist restricted to guideline content. A structured questionnaire comprising Likert-type scale items and free-text responses was distributed electronically to otolaryngologists. Quantitative responses were summarized descriptively, and thematic analysis was performed on free-text comments to identify key perceptions and concerns.

Results

Twenty-two otolaryngologists completed the survey, including attending physicians and trainees. Most respondents agreed that the checklist was accurate, clear, and safe; however, fewer indicated that it would save time or that they would be likely to use or recommend it in practice. Attending otolaryngologists more frequently endorsed checklist safety and expressed a greater willingness to use or recommend the checklist than trainees. Thematic analysis identified perceived clinical completeness and educational value as strengths, while omissions of specific examination elements were noted as limitations.

Conclusions

LLM-generated checklists derived from clinical practice guidelines were generally perceived as accurate and safe by otolaryngologists, but acceptance did not consistently translate into willingness to adopt them in practice. Perceived utility varied by level of training. These findings highlight both the potential and current limitations of LLM-generated decision-support tools and highlight the need for human oversight and further evaluation before routine clinical implementation.

## Introduction

Despite the availability of high-quality clinical practice guidelines, real-world uptake and consistent application remain variable [1]. This contributes to an implementation gap between guideline publication and bedside application, particularly in clinical environments characterized by increasing diagnostic complexity and time pressures.

These challenges are further compounded by the rapid expansion of clinical knowledge over recent decades [2], leading to frequent changes, progressive growth, and longer clinical practice guidelines. Indeed, recent studies have identified complexity, lack of conciseness [3], and lack of time [4] as contributors to decreased utilization of clinical practice guidelines by healthcare providers.

Atul Gawande popularized the use of checklists in medicine with his canonical work “The Checklist Manifesto” [5], which helped establish checklists as tools to improve reliability, standardization, and patient safety in complex clinical environments. Since then, checklists have been adopted across a range of healthcare settings, most notably in perioperative care [6] and patient safety initiatives [7,8], as mechanisms to support consistent application of best practices.

AI has increasingly been integrated into healthcare in recent years [9,10]. Large language models (LLMs), a subset of AI, use deep learning techniques to process and generate natural language responses to human input and have been proposed as tools for synthesizing and restructuring large volumes of clinical information into decision-support formats [11,12].

In the field of otolaryngology, LLMs have been tested for their utility in answering patient questions and synthesizing information. A recent study showed ChatGPT-4 produced clear, accurate, and relevant responses to commonly asked questions regarding tympanoplasty [13]. In contrast, another study reported comprehensive but occasionally erroneous information on common questions about otosclerosis [14]. However, there is little attention towards using LLMs to synthesize information for clinicians.

This study aimed to generate a structured clinical checklist from otolaryngology guidelines using an LLM and to evaluate clinician perceptions of its accuracy, usability, safety, and likelihood of adoption.

The American Academy of Otolaryngology-Head and Neck Surgery Clinical Practice Guideline: Evaluation of the Neck Mass in Adults [15] was selected as a test case for this study because it provides a rigorous framework for evaluating LLM performance in generating clinical checklists. The guideline requires the synthesis of detailed historical features, physical examination findings, and risk stratification for malignancy, and it includes safety-critical elements such as airway assessment and indications for urgent investigation. Additionally, the guideline is presented in a narrative format rather than as a structured checklist, making it suited to assessing an LLM’s ability to extract and organize clinically relevant information without introducing omissions or unsupported recommendations.

## Materials and methods

Checklist generation

The American Academy of Otolaryngology-Head and Neck Surgery (AAO-HNS) guidelines [15] were obtained and provided in full as a PDF to an LLM, ChatGPT version 5.2 (OpenAI, San Francisco, CA, USA), along with predefined written instructions (Appendix 1). These instructions directed the model to generate a concise, structured clinical checklist strictly limited to the content of the supplied guideline, explicitly excluding recommendations, interpretations, or additions not present in the original document.

The intended scope of the output was restricted to elements of clinical history and physical examination. The checklist produced by the model was subsequently transcribed into text (Appendix 2). Formatting modifications were limited to indentation and section headings to preserve the logical structure without altering the content.

Questionnaire design and distribution

A structured questionnaire (Appendix 3) was developed to evaluate clinician perceptions of the AI-generated checklist. The survey comprised a combination of Likert-type scale items and free-text responses.

The first section collected respondents' characteristics, including current training status, subspecialty, and, where applicable, years since training completion. The second section assessed baseline clinical practice patterns, including guideline use and the use of structured clinical tools. Respondents were then presented with the LLM-generated checklist and asked to rate a series of statements on a 5-point Likert-type scale (from strongly disagree to strongly agree) to evaluate clarity, completeness, perceived clinical utility, and likelihood of use in practice. The final section solicited free-text responses regarding perceived safety, limitations, and factors influencing the likelihood of using or recommending the checklist in clinical practice.

The survey was distributed electronically to otolaryngologists in the USA via social media and email from professional society lists over a three-month period. Participation was voluntary and anonymous. The University of California San Diego Institutional Review Board issued approval 814415. Descriptive statistics were used to summarize survey responses, and Fisher’s exact test was applied for comparisons where appropriate. A p < 0.05 was considered statistically significant.

## Results

Quantitative analysis

A total of 22 responses were received, with 10 (45.4%) respondents being attending otolaryngologists and 12 (54.6%) being otolaryngologists in training. Among those who had completed training, there was a fair spread across different subspecialities and lengths of independent practice (Table 1).

**Table 1:** Training level of respondents and subspecialties of attendings

Characteristic	Number
Attending otolaryngologists	10
Facial plastics and reconstructive surgery	1
Laryngology	2
Rhinology	1
Otology and neurotology	4
Head and neck oncology	1
General otolaryngology	2
Fellow in subspecialty	1
Resident in otolaryngology	11

**Figure 1:**
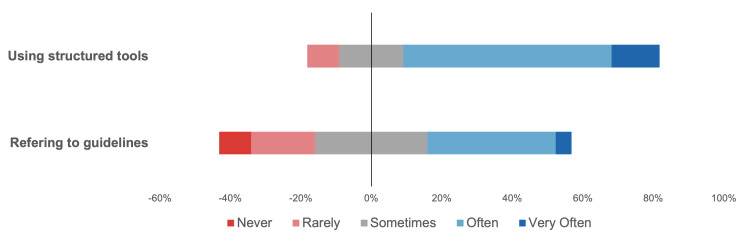
Self-reported baseline use of guidelines and structured tools

Most respondents (n = 16, 72.7%) reported referring to guidelines sometimes or more frequently, while a minority (n = 6, 27.3%) reported rarely or never using guidelines. Interestingly, five of 11 otolaryngologists who had completed residency reported rare or no use of guidelines, compared with 0 residents reporting the same (p = 0.035). Twenty (90.9%) of the respondents reported using structured tools sometimes or more frequently (Figure 1).

**Figure 2:**
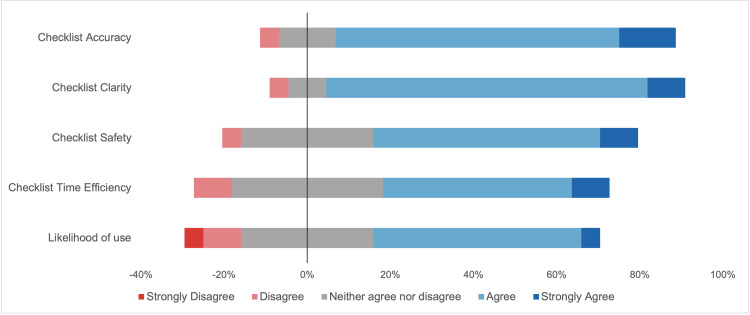
Clinician perceptions of the AI-generated checklist AI: artificial intelligence

When evaluating the checklist, 18 (81.8%), 19 (86.3%), and 14 (63.6%) respondents agreed or strongly agreed that the checklist was accurate, clear, and safe, respectively (Table 2). However, only 12 (54.5%) participants agreed or strongly agreed that use of the checklist would save time or that they would be likely to use it in their practice (Figure 2).

**Table 2:** Distribution of clinician responses to checklist evaluation statements

This checklist is…	Strongly disagree	Disagree	Neither agree nor disagree	Agree	Strongly agree
Accurate and consistent	0	1	3	15	3
Clear and easy	0	1	2	17	2
Safe to use	0	1	7	12	2
Time efficient	0	2	8	10	2

When stratified by level of training, eight (80%) attending otolaryngologists endorsed the checklist as safe, compared with six (50%) otolaryngologists in training. Differences were also observed in the likelihood of use or recommendation, with seven (70%) attendings agreeing or strongly agreeing to use or recommend the checklist, compared with five (41.7%) residents.

Thematic analysis

Themes identified from the qualitative analysis of free-text responses are summarized in Table 3.

**Table 3:** Themes identified from qualitative analysis of free-text responses

Theme	Relevant quotes
Clinical completeness	"Touches on all pertinent signs and symptoms that could lead to neck mass"
	"Helpful for residents seeing consults to ensure that the important things are evaluated and captured in a note"
	"The checklist is concise, easy to read with pertinent high yield points that will assist all providers taking care of the patient."
	"Very thorough and itemized"
Efficiency	“Time saving”
	“Ease of use. Availability”
	“How easy to integrate to EHR”
	“Simple, straightforward”
Differential usefulness by training level	“Probably a good checklist for a non otolaryngologist”
	“Helpful for residents seeing consults”
	“Would be helpful for a medical student or early resident”
	“Good for general or primary medical doctors but not needed for ENT specialists”
	“After training, this checklist does not add or contribute significantly”
Missing information	"Airway symptoms"
	"Scope is typically part of ENT physical exam for neck masses"
	"Family history, scope exam"

Clinical completeness

Many respondents testified to the checklist's completeness, with one stating that the checklist “…touches on all pertinent signs and symptoms…". Responses framed this clinical completeness as both a cognitive aid and a documentation support tool, with utility perceived for use during responses to consults.

Differential utility by training level

A recurring theme among free-text responses was the differential utility of the checklist across training levels. The checklist was widely viewed as most valuable for clinicians earlier in training and for non-otolaryngologists, with some otolaryngologists referring to established frameworks acquired through training, noting that they “…already have this checklist in (their) head…”.

Important omissions

One of the questions sought to elicit perceived missing information from respondents. Three key elements were mentioned as not included in the generated checklist, despite being of considerable importance in the workup of a neck mass: the inclusion of a “scope” as part of the examination, family history, and airway-related symptoms. Notably, all of these components are included in the source AAO-HNS clinical practice guideline [15] but were not captured in the checklist generated by the language model. Notwithstanding this theme, only one respondent disagreed with the description of this checklist as safe.

## Discussion

To our knowledge, this study is among the first to assess an LLM's ability to extract a concise checklist from otolaryngology clinical practice guidelines and to evaluate its output through structured clinician feedback. This study showed that an LLM can generate a checklist when fed a guideline, which is largely acceptable to otolaryngologists; acceptance does not equate to universal willingness to adopt, and perceived utility varies by training level.

Our study showed that a majority of otolaryngologists found the generated checklist accurate and clear. However, just over half of respondents reported they would use or recommend this checklist, despite an overwhelming majority endorsing the use of structured tools in their current practice. This could be attributable in part to respondents expressing that their training equips them with the knowledge and habits to already ask these questions.

Respondents suggested that the checklist could be used as an educational tool rather than implemented into their workflow, citing its utility for medical students and junior residents. Differences in perceptions by level of training were observed, with attending otolaryngologists more frequently endorsing the checklist as safe and expressing greater willingness to use or recommend it compared with trainees. Differences by level of training may also reflect greater adaptability among attending otolaryngologists, who may be more accustomed to integrating adjunctive decision-support tools into established clinical workflows and exercising clinical judgment in their application.

When evaluating the generated checklist, a few important omissions were identified, including laryngoscopy as part of the physical exam, airway symptoms, and family history. Interestingly, despite these important omissions, none of the responses suggested the checklist was unsafe, possibly due to the underlying assumption of concurrent clinician oversight in its use. A recent study by Singhal et al. [16] demonstrated that even LLMs adapted and trained for medical applications did not consistently match clinician performance in terms of correctness, underscoring the continued need for clinical oversight when such systems are used in healthcare.

Importantly, on review of our checklist, we did not find any unsafe recommendations or “hallucinations.” LLMs are known to make errors in document summarization [17] and clinical tasks [18,19], prompting caution regarding their use in healthcare. Hallucinations [20] are defined as false information generated by the LLM that is not present in the input or source data. We hypothesize that this was in part due to our detailed prompt explicitly instructing the LLM not to report any data that is not present in the input. Indeed, hallucinations have been associated with a prompting strategy [21]. A previous study by Kıyak et al. [22], which sought to evaluate the utility of LLMs in generating exam-style questions from clinical practice guidelines, also noted that no output required major correction due to the structured input.

Human evaluation is recognized as essential for assessing the safety and clinical relevance of LLM outputs in healthcare [21]. Similar to our survey, prior studies evaluating LLMs in healthcare commonly employ Likert-scale instruments to assess the quality of output [23,24].

The limitations of our study include the use of a single complex, text-heavy guideline, which means the findings may not be generalizable to other guidelines with different structures or levels of complexity. The sample size was also modest and included only otolaryngologists, which limits generalizability to other healthcare professionals, such as primary care physicians or physician assistants.

A recent study by Aliyeva et al. [13] found that Claude 3.5 Sonnet (Anthropic, CA, USA) outperformed ChatGPT-4 in providing advice on commonly asked questions from patients following tympanoplasty. Our study evaluated only one specific LLM's performance, and the checklist generated could differ between versions and LLMs used. Lastly, our survey evaluated perceived utility rather than real-life use, without measurement of objective patient-linked parameters as outcomes.

Future work should evaluate LLM-generated checklists across multiple guidelines and assess their impact on documentation quality, diagnostic accuracy, and workflow efficiency in real-world clinical settings.

## Conclusions

LLMs can extract guideline content into structured clinical checklists that are generally perceived as accurate and safe by otolaryngologists. However, perceived acceptability does not consistently translate into willingness to adopt in practice, with utility varying by training level and concerns regarding completeness remaining. These findings suggest LLM-generated decision-support tools may serve an adjunctive educational or cognitive-aid role but require human oversight and further validation before routine clinical implementation.

## Appendices

**Table 4:** Appendix 1: Prompt provided to ChatGPT version 5.2 (OpenAI, San Francisco, CA, USA) for checklist generation

You are an otolaryngologist	
Using the following clinical guideline text, generate a concise, structured checklist of key history and physical examination elements that should be assessed during the initial outpatient evaluation of an adult patient with a neck mass.	
Requirements:	
	Include only items explicitly stated or clearly implied in the guideline
	Organize the checklist into logical sections (e.g., history, physical examination, red flags)
	Where appropriate, indicate conditional logic (e.g. “if present, consider escalation”)
	Do NOT include investigations, imaging, laboratory tests, or management decisions
	Do NOT add recommendations not supported by the guideline
	Use clear, clinician-friendly language suitable for real-world outpatient practice
Output format:	
	Bullet-point checklist
	No prose explanations
	No references

**Table 5:** Appendix 2: Checklist generated by ChatGPT version 5.2 (OpenAI, San Francisco, CA, USA)

Section	Item	Details
History	Duration of neck mass	Present ≥2 weeks without significant fluctuation → increased risk
History	Duration of neck mass	Duration uncertain → increased risk
History	Infectious history	Recent URI, dental infection, trauma (incl. insect bite), travel, animal exposure
History	Infectious history	If no infectious history → increased risk
History	Pain/tenderness	Nontender mass → increased risk
History	Age	≥40 years → increased risk
History	Risk factors	Tobacco use
History	Risk factors	Alcohol use
History	Risk factors	Immunocompromised status
History	Head and neck cancer–related symptoms	Pharyngitis or throat pain
History	Head and neck cancer–related symptoms	Dysphagia or odynophagia
History	Head and neck cancer–related symptoms	Hoarseness or recent voice change
History	Head and neck cancer–related symptoms	Otalgia ipsilateral to mass
History	Head and neck cancer–related symptoms	Hearing loss ipsilateral to mass
History	Head and neck cancer–related symptoms	Nasal obstruction or unilateral epistaxis
History	Head and neck cancer–related symptoms	Hemoptysis or blood in saliva
History	Head and neck cancer–related symptoms	Oral cavity or oropharyngeal ulcer/swelling
History	Head and neck cancer–related symptoms	Unexplained weight loss
Past history		Prior head and neck malignancy (including skin, salivary gland, aerodigestive tract)
Past history		Lymphoma-related symptoms
Past history		Fever, night sweats, weight loss
Past history		Lymphadenopathy outside head and neck
Physical examination	Neck mass characteristics	Size >1.5 cm → increased risk
Physical examination	Neck mass characteristics	Firm consistency → increased risk
Physical examination	Neck mass characteristics	Reduced mobility/fixation to adjacent tissues → increased risk
Physical examination	Neck mass characteristics	Ulceration of overlying skin → increased risk
Physical examination	Neck mass characteristics	Tender vs nontender (nontender more suspicious)
Physical examination	Neck mass characteristics	Multiple, grouped, or matted nodes
Physical examination	Skin examination	Face, neck, scalp: ulcerations or pigmented lesions
Physical examination	Skin examination	Oral cavity
Physical examination	Skin examination	Inspect all surfaces (remove dentures)
Physical examination	Skin examination	Palpate floor of mouth
Physical examination	Skin examination	Assess tongue mobility
Physical examination	Oropharynx	Tonsillar asymmetry
Physical examination	Oropharynx	Ulcer or mass (palate, tonsils, posterior pharyngeal wall)
Physical examination	Oropharynx	Palpation of tonsils and base of tongue (as feasible)
Physical examination	Neck and thyroid	Palpate thyroid gland and surrounding neck structures
Red flags/increased risk for malignancy	Standalone feature	No infectious etiology
Red flags/increased risk for malignancy	Standalone feature	Mass ≥2 weeks or uncertain duration
Red flags/increased risk for malignancy	Standalone feature	Size >1.5 cm
Red flags/increased risk for malignancy	Standalone feature	Firm, fixed, or reduced mobility
Red flags/increased risk for malignancy	Standalone feature	Skin ulceration
Red flags/increased risk for malignancy	Standalone feature	Any additional suspicious history or exam finding

**Table 6:** Appendix 3: Questionnaire used to evaluate the AI-generated clinical checklist AI: artificial intelligence Generated using ChatGPT version 5.2 (OpenAI, San Francisco, CA, USA)

Section	Question	Response options
Demographics	1. What is your current role?	Attending otolaryngologist; Fellow in otolaryngology subspecialty; Resident in otolaryngology
Demographics	2. What is your primary subspecialty?	General otolaryngology; Rhinology; Otology & Neurotology; Head & Neck Oncology; Laryngology; Facial Plastic & Reconstructive Surgery; Paediatric otolaryngology
Demographics	3. How long have you been out of training?	0 years; 1–5 years; 5–10 years; More than 10 years
Baseline clinical practice	4. How often do you refer to clinical practice guidelines (e.g., AAO-HNS, EPOS, NCCN) in routine decision-making?	Never; Rarely; Sometimes; Often; Very often
Baseline clinical practice	5. How often do you use structured tools (checklists, algorithms, EHR templates/smart phrases)?	Never; Rarely; Sometimes; Often; Very often
Checklist evaluation	6. The checklist is clinically accurate and consistent with current guideline recommendations.	Strongly disagree; Disagree; Neither agree nor disagree; Agree; Strongly agree
Checklist evaluation	7. The checklist is clear and easy to understand.	Strongly disagree; Disagree; Neither agree nor disagree; Agree; Strongly agree
Checklist evaluation	8. The checklist would be safe to use as clinical decision support, with appropriate clinician oversight.	Strongly disagree; Disagree; Neither agree nor disagree; Agree; Strongly agree
Checklist evaluation	9. Use of this checklist would save time compared with consulting the full guideline text.	Strongly disagree; Disagree; Neither agree nor disagree; Agree; Strongly agree
Checklist evaluation	10. I would be likely to use or recommend this checklist if integrated into my clinical workflow.	Strongly disagree; Disagree; Neither agree nor disagree; Agree; Strongly agree
Open-ended	11. Please briefly explain the factors that influenced your likelihood of using or recommending this checklist in clinical practice.	(Free-text response)
Open-ended	12. Did you identify any important missing items from the checklist?	(Free-text response)
Open-ended	13. Did you identify any potential incorrect or unsafe recommendations?	(Free-text response)
